# Design, implementation and evaluation of non-contact in vivo confocal microscopy for capturing the human corneal endothelium from the central to near-limbal regions

**DOI:** 10.1016/j.zemedi.2025.12.001

**Published:** 2025-12-18

**Authors:** Karsten Sperlich, Alois Gottschlich, Karsten Winter, Florian Worsch, Oliver Stachs, Sebastian Bohn

**Affiliations:** a Department of Ophthalmology, Rostock University Medical Center, 18057 Rostock, Germany; b Department Life, Light & Matter, University of Rostock 18059 Rostock, Germany; c Institute of Anatomy, University of Leipzig 04103 Leipzig, Germany

## Abstract

**Purpose:**

Current imaging techniques for assessing the human corneal endothelium are constrained by drawbacks related to corneal contact, imaging artifacts, and a restricted field of view (FOV). We present a non-contact in vivo confocal microscopy (IVCM) configuration enabling cellular-resolution endothelial imaging.

**Methods:**

A commercially available scanning laser ophthalmoscope was modified with a long-working-distance microscope objective enabling non-contact IVCM of the corneal endothelium. Central endothelial images were obtained in one healthy volunteer using the novel non-contact IVCM and two established modalities: contact IVCM and specular microscopy. A cell segmentation algorithm was applied to compare modalities. For the non-contact IVCM, near-limbal images were obtained in a second volunteer demonstrating far-peripheral imaging. For demonstration purposes, additional five healthy volunteers were imaged.

**Results:**

The non-contact IVCM achieved a lateral resolution below 2.2 µm, while offering a larger FOV. Obtained quantitative metrics - including endothelial cell density, mean cell area, and polygonality - were consistent across modalities. The system also enabled high-quality imaging of peripheral and near-limbal endothelial regions with resolution and contrast similar to central areas.

**Conclusion:**

This non-contact IVCM system delivers cellular-resolution imaging of the human corneal endothelium across central to near-limbus without topical anesthesia or corneal contact. It preserves the quantitative consistency of contact confocal microscopy yet offers greater comfort and a larger sampling area. To our knowledge, the herein presented IVCM is currently the only non-contact, high-resolution imaging modality demonstrating endothelial imaging in the limbal region in vivo. These results offer a patient-friendly alternative for routine quantitative endothelial assessment.

## Introduction

The corneal endothelium is a monolayer of hexagonally arranged cells on the posterior surface of the cornea that maintains stromal deturgescence and optical transparency through barrier and pump functions [1]. Because of its limited regenerative capacity in vivo [2], persistent or pathologically accelerated cell loss can, under certain conditions, lead to corneal decompensation, edema, and vision impairment. This process is particularly relevant in disorders such as Fuchs’ endothelial corneal dystrophy (FECD) [3] and pseudophakic bullous keratopathy (PBK) [4], but also in glaucoma therapy and especially glaucoma stents [5,6].

Current clinical evaluation of the endothelium relies on slit-lamp biomicroscopy, specular microscopy, and contact confocal microscopy, each with characteristic trade-offs. Slit-lamp–based specular reflection is a non-contact, first-line method for visualizing the corneal endothelium, but its limited resolution and field of view (FOV), operator dependence and sensitivity to media opacities [7], restrict reliable quantitative analysis. Dedicated non-contact specular microscopes (e.g., EM-4000, Tomey Corporation, Nagoya, Japan; CellCheck20+, Konan Medical Inc., Irvine, USA, or CEM-530, Nidek Co. Ltd., Gamagori, Japan) improve resolution and allow (semi-)automated determination of endothelial cell density and morphology.

The FOV remains small for all non-contact specular microscopes, measuring approximately 0.135 mm^2^ for the Tomey EM-4000 [personal communication with manufacturer], 0.1375 mm^2^ for the Konan CellChek 20 PLUS [8], and 0.1375 mm^2^ for the NIDEK CEM-530 [9], respectively. Image acquisition in these systems is based on discrete fixation targets rather than a continuous scan. The EM-4000 captures images at 13 predefined corneal locations, one central and six paracentral positions approximately 0.55 mm from the corneal center, and six peripheral positions up to about 3.5 mm [personal communication with manufacturer]. The CellChek 20 PLUS acquires 1 central and 12 paracentral images at ≈ 3.0 mm (6 points), ≈ 4.0 mm (4 points), and ≈ 4.5 mm (2 points) from the center [8], while the CEM-530 acquires one central, eight paracentral (≈ 0.65 mm), and six peripheral (≈ 3.65 mm) locations [9], respectively. Despite these multiple fixation points, the limited per-frame sampling area (≈ 0.1375 mm^2^) results in insufficient overlap between adjacent fields, precluding the generation of continuous wide-field endothelial mosaics. Consistent with prior reviews, current non-contact specular microscopes are limited to central and mid-peripheral corneal imaging and cannot reliably capture near-limbal endothelium [10]. For this study the zonal definitions (radii from corneal center) are: central ≤ 2.0 mm; paracentral > 2.0 - 5.0 mm; peripheral > 5.0 mm; near-limbal ≥ 5.5 mm. These definitions were adopted to ensure consistency across devices, as manufacturers use slightly different zonal specification.

Contact in vivo confocal microscopy (IVCM) achieves higher spatial resolution with approximately 1 µm lateral and 4 µm axial resolution [11] but retains a small FOV similar to specular microscopy [12], so broader assessment typically requires multi-site sampling or mosaicking [13]. Because it requires topical anesthesia and applanation with a sterile contact cap [14], it remains minimally invasive and operator-dependent. Excessive applanation pressure is known to induce compression artefacts, particularly in subbasal nerve plexus imaging, underscoring the need for careful operator control during contact confocal microscopy [15].

Unlike non-contact specular microscopy systems, which are limited to the central and paracentral zones [10], our approach enables imaging of the peripheral endothelium without physical contact, surpassing FOV constraints seen in both specular and confocal modalities. Peripheral endothelial cell counts have been shown to correlate strongly with disease severity in advanced Fuchs’ endothelial corneal dystrophy, making peripheral imaging diagnostically valuable [16]. In line with this, we included near-limbal fields to capture regional endothelial cell loss, that may be missed in central and paracentral imaging. To address the limitations of existing methods, we developed a non-contact confocal system that preserves cellular-level resolution, improves patient comfort, and acquires high-resolution images from central to far-peripheral endothelium, enabling large, spatially representative datasets that reduce sampling bias and support earlier detection of localized or asymmetric pathology.

## Methods

**Fig. 1:**
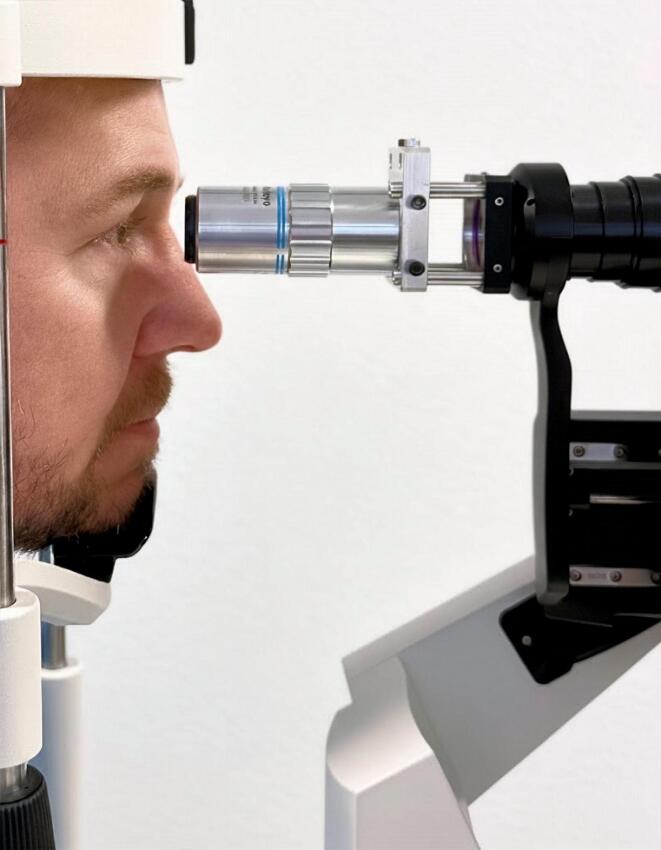
Setup for the non-contact confocal in vivo imaging of the corneal endothelium. Subject in head and chin rest (left); Mitutoyo M Plan objective lens (50x, NA = 0.42, working distance = 20.5 mm, visible light) mounted in a custom adapter and with cage rods to a modified cage plate for clamping on the HMM (center); Modified SPECTRALIS OCT2 with attached HMM (right)

To avoid the general complications associated with contact corneal imaging techniques, e.g. topical anesthesia, infection risk, patient compliance and contact-dependent imaging, and to overcome the constraints of the limited FOV, we developed a non-contact confocal imaging setup using a modified scanning laser ophthalmoscope (SPECTRALIS HighRes OCT, Heidelberg Engineering, Germany). Throughout the manuscript, we will use the term non-contact IVCM for the setup shown in Fig. 1. The system provides multiple illumination wavelengths which can be used for confocal (481 nm, 513 nm, 725 nm) or OCT (834 nm ± 141 nm) imaging [17]. To shift the focal plane from the retina to the posterior corneal surface while maintaining confocal alignment, a 50x microscope objective lens with super long working distance (20.5 mm) and a numerical aperture of 0.42 optimized for visible light (M Plan Apo SL 50x / 0.42, Mitutoyo Corporation, Kawasaki, Japan) was mounted to a High Magnification Module (HMM, Heidelberg Engineering GmbH, Heidelberg, Germany), which was attached to the ophthalmoscope. The distance between microscope objective lens and HMM was adjusted such that the exit pupil of the microscope objective lens was in the scan pupil of the ophthalmoscope with attached HMM. For endothelial imaging, the green illumination channel (513 nm) was selected, offering a good balance between intrinsic contrast of the endothelial layer and reflectivity. For example, using near-infrared light, the endothelium did not offer sufficient intrinsic contrast while green and blue resulted in similar image quality and representation of structural details as shown in [18] with another objective lens in a contact approach.

The HighSpeed mode was used, providing 15 frames per second with an image size of 768 x 768 pixels. The lateral magnification was determined from a known distance of a 1951 United States Air Force (USAF) resolution target image (#38-257, Edmund Optics, Barrington, NJ, USA). The lateral optical resolution is defined as the line width of the largest still blurred USAF resolution target element.

The optical output power was measured using an optical power meter (1918-R, Newport Corporation, Irvine, CA, USA) with a diode based detector head (918D-UV-OD3R, Newport Corporation, Irvine, CA, USA).

To evaluate the performance of the non-contact imaging approach, we conducted comparative endothelial imaging in a healthy male volunteer (age: 41 years) with no history of ocular diseases, surgery, or contact lens wear. The right eye was examined using specular microscopy (Tomey EM-4000), contact IVCM, and the novel non-contact IVCM setup described above. To ensure that the non-contact imaging techniques remained unaffected, contact IVCM was performed as the final step of the examination protocol. For the demonstration of near-limbal endothelium, the right eye of a 38-year-old healthy male volunteer was investigated.

Specular microscopy was performed according to the manufactureŕs standard protocol. Several images of the central corneal endothelium were acquired, and the frame with the highest image quality and well-defined cell borders was selected for further analysis.

Contact IVCM was performed using a confocal laser scanning ophthalmoscope Heidelberg Retina Tomograph II (HRT2, Heidelberg Engineering GmbH, Germany) equipped with Rostock Cornea Module (RCM, Heidelberg Engineering GmbH, Germany). After the instrument’s camera was retracted, the subject was positioned with the chin and forehead resting on the standard headrest assembly. A sterile single-use TomoCap (Tomocap, Heidelberg Engineering GmbH, Germany) with a sterile coupling gel (Vidisic, Bausch und Lomb GmbH, Berlin, Germany) applied on both sides was used for applanation of the corneal surface after application of topical anesthesia on the cornea. Multiple scans of the central endothelium were acquired. From these, the image with the apparently highest contrast and well delineated cell borders was selected for further analysis.

For non-contact IVCM the subject was positioned with the chin and forehead resting on the standard headrest support. Care was taken that the instrument’s camera was retracted allowing a sufficient clearance between the objective lens and the corneal apex. Imaging was performed in reflection mode with the green illumination channel active. For regions close to the limbus, imaging was achieved by having the contralateral eye fixate a temporal or nasal target, shifting the FOV toward the corresponding peripheral endothelium. Once the limbal margin was identified, the instrument was moved slightly backward, to shift the focal plane onto the limbal endothelium. In cases of restricted ocular motility, the subject may fixate on an alternate, stationary point and the non-contact IVCM platform can be rotated to image the desired peripheral area entirely eliminating the need for head or eye movement.

A custom software, implemented in Python (Python Software Foundation, Wilmington, DE, USA), was used to allow continuous recording of the confocal images. During steady fixation, the operator manually adjusted the focus through the corneal stroma until the endothelial cells became visible. A short continuous image sequence (approximately 10 s to 30 s) was acquired at the endothelium for each subject and the image with evenly illuminated endothelium and well-defined cell borders was selected for further processing. The total examination time was about 1 min to 3 min per subject.

For comparative image analyses between the imaging modalities without bias from a dedicated algorithm, quantitative and morphological characterization of the corneal endothelium was performed using a custom algorithm implemented in Mathematica (Wolfram Research Inc., Champaign, IL, USA) and originally developed for epithelial analyses [19]. It is fully automated and does not require any individual preprocessing steps. The same algorithm was applied to all images to ensure a uniform and unbiased evaluation of the different modalities. It automatically detected and segmented individual cells and computed key parameters, including cell count, density, polygonality, number of neighbors, mean diameter and mean area.

## Results

Regarding the novel method presented here, the lateral magnification was determined to 0.7066 µm/pixel using a USAF resolution target. The image size of the HighSpeed mode is 768 x 768 pixels resulting in a FOV of 0.295 mm^2^.

The highest achievable lateral optical resolution could not be determined exactly, as even the smallest element (Group 7, Element 6) of the USAF resolution target was clearly resolved. Therefore, we can only state that the optical resolution is better than 2.2 µm.

The optical output power impinging on the corneal surface (30 µW over 0.295 mm^2^; ≈ 10 mW/cm^2^) was well below the International Commission on Non-Ionizing Radiation Protection (ICNIRP) [20] and American National Standards Institute (ANSI) [21] maximum permissible exposure limits for visible continuous-wave light. As the beam is focused on the cornea and diverges within the eye, retinal hazard is negligible and the illumination complies with Class 2 eye-safety standards according to International Electrotechnical Commission (IEC) 60825-1 [22].

**Fig. 2:**
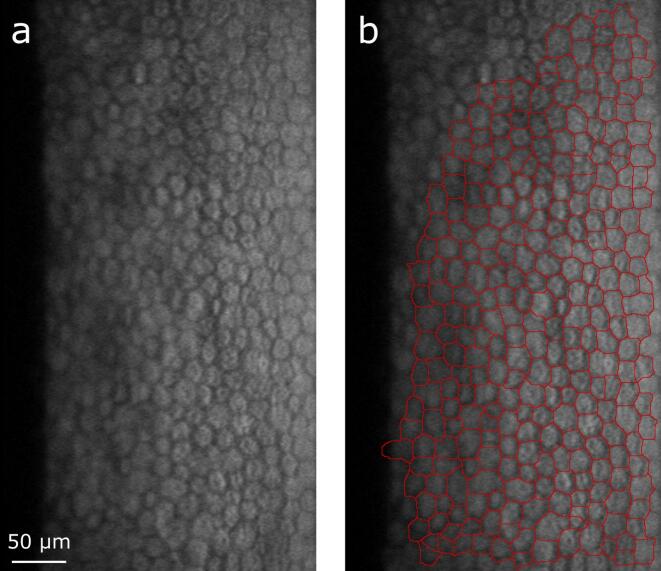
Specular microscopy of the corneal endothelium acquired with the EM-4000: (a) original image and (b) the same image with automated cell segmentation (red overlay). The FOV was 0.135 mm^2^ and the segmented area was 0.110 mm^2^ (81.5 % of FOV).

In all three image modalities, the corneal endothelium of the 41-year-old male volunteer could be visualized, demonstrating cellular-level imaging capability (cf. Fig. 2a to Fig. 4a). For better comparability, Fig. 2 to Fig. 4 have the same scale. Additionally, Fig. 2b to Fig. 4b present the same image with the cell segmentation overlay.

Comparing image appearance, specular microscopy exhibited good sharpness in the central vertical region but provided limited field coverage, with reduced contrast and sharpness toward the lateral periphery (see Fig. 2).

**Fig. 3:**
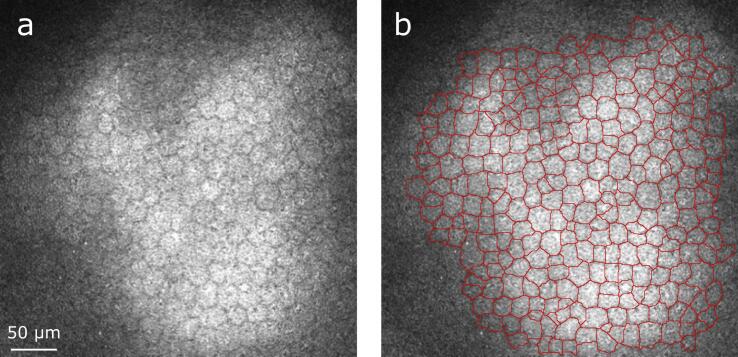
Contact in vivo confocal microscopy of the corneal endothelium acquired with the HRT2/RCM: (a) original image and (b) the same image with automated cell segmentation (red overlay). The FOV was 0.160 mm^2^ and the segmented area was 0.100 mm^2^ (62.5 % of FOV).

Contact IVCM provided cellular-level details within a limited FOV. However, cell borders were not uniformly distinct across the frame, and illumination was uneven. Peripheral regions within the image appeared blurred or poorly resolved, resulting in incomplete visualization of endothelial cells across the entire image (see Fig. 3).

**Fig. 4:**
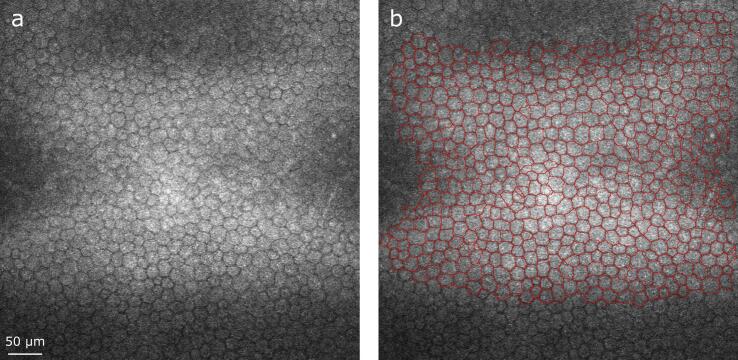
Non-contact in vivo confocal microscopy of the corneal endothelium acquired with the modified SPECTRALIS setup: (a) original image and (b) the same image with automated cell segmentation (red overlay). The FOV was 0.295 mm^2^ and the segmented area was 0.190 mm^2^ (64.4 % of FOV).

The non-contact scanning laser ophthalmoscopy (SLO) image exhibited a larger FOV and more uniform illumination compared to the other modalities. Endothelial cell borders were well delineated across most of the frame, including some peripheral regions, indicating consistent image quality throughout the field (cf. Fig. 4). However, an even larger FOV would presumably suffer from the natural curvature of the endothelium.

**Fig. 5:**
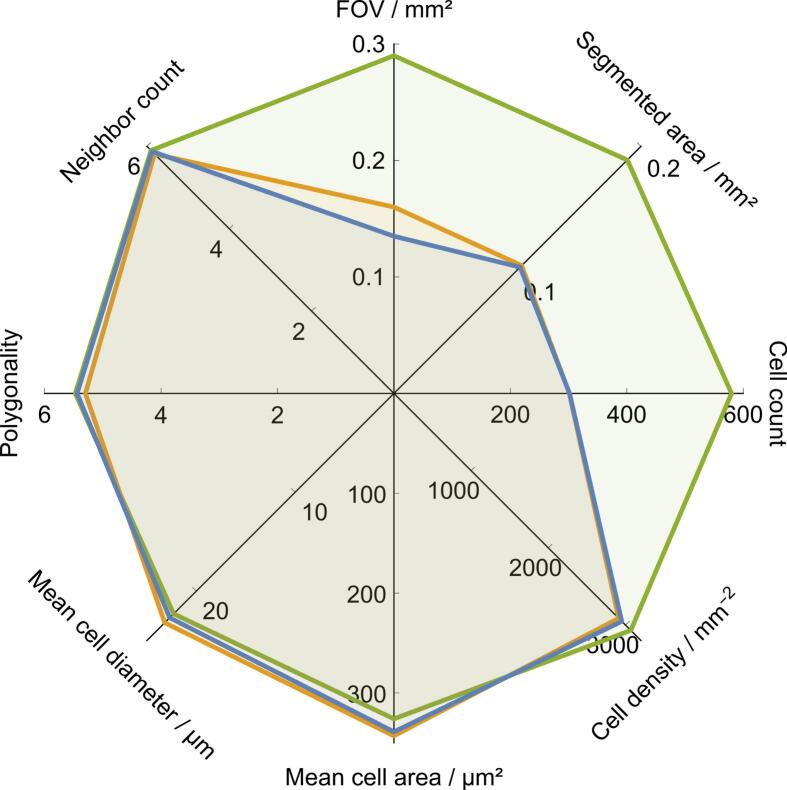
Radar chart comparing morphometric parameters of the 41-year-old male volunteer across the modalities. Neighbor count, polygonality, mean cell diameter, area and density were similar for all three methods (blue: specular microscopy; orange: contact IVCM; green: novel non-contact IVCM). In contrast, the non-contact IVCM showed markedly higher field of view, segmented area, and absolute cell count.

Beyond visual appearance, quantitative parameters were analyzed to compare imaging performance and segmentation outcomes across modalities. Fig. 5 summarizes the quantitative comparison of imaging performance across the three modalities. The radar chart illustrates both the FOV and the successfully segmented area, along with the morphometric parameters derived from automated cell segmentation using the algorithm presented in [19]. Parameters (values given order: specular microscopy, contact IVCM, and non-contact IVCM) such as mean cell diameter (22.8 µm, 23.2 µm, 22.3 µm), mean cell area (340 µm^2^, 343 µm^2^, 326 µm^2^), and endothelial cell density (2944 cells/mm^2^, 2912 cells/mm^2^, 3069 cells/mm^2^) were similar across all modalities, as expected for measurements acquired centrally from the same eye of the same healthy subject. Likewise, mean polygonality (5.5, 5.3, 5.5) and mean neighbor count (5.9, 5.8, 5.9) showed minimal variation between methods.

While specular microscopy and contact IVCM yielded similar values for all parameters, the non-contact IVCM approach provided the largest FOV measuring 0.295 mm^2^ which is approximately 2.16 times larger than specular microscopy (0.135 mm^2^) and 1.83 times larger than the IVCM frame (0.160 mm^2^). The successfully segmented area (0.11 mm^2^, 0.10 mm^2^, 0.19 mm^2^) was also greater, resulting in a higher absolute cell count (319, 301, 580) per image.

**Fig. 6:**
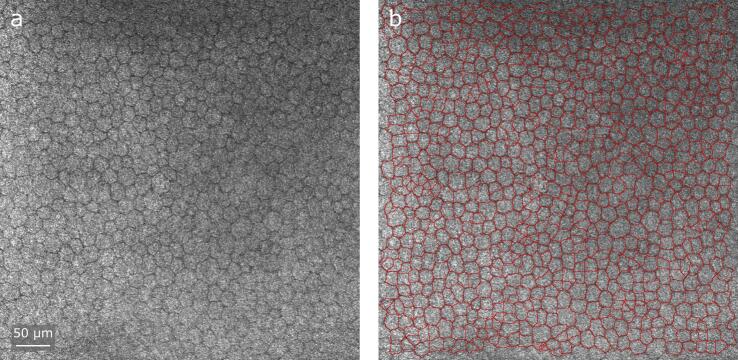
Non-contact in vivo confocal microscopy of the corneal endothelium near the temporal limbus acquired with the modified SPECTRALIS setup: (a) original image and (b) the same image with automated cell segmentation (red overlay). The FOV was 0.295 mm^2^ and the segmented area was 0.263 mm^2^ (89.2 % of FOV).

Fig. 6 shows an image of the near-limbal corneal endothelium of the 38-year-old male volunteer acquired with the non-contact IVCM setup. Despite the increased eccentricity, image quality was comparable to that of the central region, with clearly delineated endothelial cell borders and uniform illumination across the field. The field of view, resolution, and overall image characteristics remained unchanged, demonstrating that peripheral endothelial imaging can be achieved with similar image quality using the same acquisition parameters. The automated segmentation resulted in the following parameters: mean cell diameter (22.2 µm), mean cell area (316 µm^2^), endothelial cell density (3166 cells/mm^2^), polygonality (5.4), mean neighbor count (5.8) and cell count (833).

Comparing the Fig. 4, Fig. 6, intensity inhomogeneities within the endothelium can be observed. There are multiple reasons: a) neglecting aberrations, the image is a plane surface and the endothelium is an approximately spherical single cell layer b) the endothelium is not on a perfect sphere and c) the image plane maybe not tangential to the sphere. Hence, the single cell layer is out of focus for some regions. As in Fig. 6 the left and bottom side of the image exhibit stromal features, it indicates a non-tangential orientation of the image plane to the endothelium.

To demonstrate the imaging capability, we provide 5 supplementary figures (Fig. S1 to Fig. S5) of 5 additional healthy volunteers (3 male, 2 female, between 21 years to 62 years old).

In summary, the non-contact IVCM approach achieved comparable cellular resolution to conventional techniques while providing a substantially larger FOV and segmentation area, thereby enabling the analysis of higher number of endothelial cells per image.

## Conclusion

The novel non-contact IVCM system enabled reliable in vivo imaging of the human corneal endothelium. Endothelial cell borders were clearly delineated across almost the entire field of view, allowing quantitative analysis. For an unbiased morphometric cell characterization, the same algorithm was used to assess the endothelium captured by the three approaches. Key parameters, including endothelial cell density, mean cell area, and mean polygonality, showed high consistency with values obtained from all three imaging modalities. Despite differences in acquisition principle and FOV, the quantitative agreement across modalities confirms that the new approach achieves comparable cellular resolution while providing a substantially larger and more uniformly illuminated imaging field.

Additionally, the transition to a completely non-contact design eliminates the need for corneal applanation and topical anaesthesia, thereby minimizing the risk of applanation-induced imaging effects, epithelial injury, infection, or allergic reactions, while simultaneously achieving higher patient comfort. It also shortens the examination time compared with contact-based IVCM, while image acquisition itself requires a duration comparable to the EM-4000. However, the EM-4000 currently provides an integrated analysis pipeline for deriving key parameters. Furthermore, the extended field of view in the non-contact IVCM results in a higher absolute cell count per frame, approximately twice that of conventional methods, while maintaining equivalent morphometric parameters.

To our knowledge, no specular microscope is capable of imaging the endothelium in the near-limbal region, whereas the non-contact IVCM readily captures this area. Although contact IVCM can image the periphery in principle, it typically requires an experienced examiner and a highly compliant subject. In practice, the non-contact system enables endothelial imaging from the central to near-limbal cornea, providing access to endothelial morphology over a substantially larger sampling area. This expanded sampling area provides the technical basis for future work aimed at generating large-area endothelial maps. However, full-cornea endothelial maps in vivo are currently not feasible due to very long total examination durations.

Importantly, the system is based on a modified SPECTRALIS platform and remains compatible with the standard objective lenses, allowing conventional fundus imaging to be performed as usual.

While the present study demonstrates the technical feasibility and clinical potential of non-contact confocal endothelial imaging, several limitations remain. The current implementation relies on manual frame selection and analysis, which may introduce observer bias. Automated frame selection - maybe even combined with real-time segmentation - will be the essential next steps to enable routine clinical application.

While several subjects were examined using the novel setup to demonstrate the feasibility, the direct device comparison presented herein was limited to a single healthy volunteer. To illustrate imaging the capabilities across individuals, representative endothelial images from additional healthy volunteers are provided in the supplementary material. Validation across a larger cohort, including eyes with corneal pathologies, is required to assess robustness under clinical conditions and to determine diagnostic sensitivity in diseased corneas. An algorithm dedicated to endothelial cell characterization should be implemented as well for direct comparison to commercially available endothelial imaging devices. Furthermore, a precise calibration of the effective field of view and magnification correction should be implemented to ensure accurate absolute measurements of endothelial cell density possible also depending on the corneal thickness.

These results provide a strong basis for clinical and research applications, showing that non-contact in vivo confocal imaging is able to deliver comprehensive, high-quality visualization of the human corneal endothelium with greater patient comfort and diagnostic utility.

## CRediT authorship contribution statement

**Karsten Sperlich:** Writing – review & editing, Writing – original draft, Visualization, Methodology, Funding acquisition, Data curation, Conceptualization. **Alois Gottschlich:** Writing – review & editing, Writing – original draft, Software, Investigation. **Karsten Winter:** Writing – review & editing, Writing – original draft, Visualization, Software, Formal analysis. **Florian Worsch:** Writing – review & editing, Writing – original draft, Software. **Oliver Stachs:** Writing – review & editing, Supervision, Project administration, Conceptualization. **Sebastian Bohn:** Writing – review & editing, Supervision, Project administration, Investigation.

## Declaration of competing interest

The authors declare that they have no known competing financial interests or personal relationships that could have appeared to influence the work reported in this paper.
