# Supplementary material for: Design, implementation and evaluation of non-contact in vivo confocal microscopy for capturing the human corneal endothelium from the central to near-limbal regions
Source: Z Med Phys. 2025 Dec 18;36(2):144–50. doi: 10.1016/j.zemedi.2025.12.001 (PMC13316533; doi:10.1016/j.zemedi.2025.12.001)
Supplement: Supplementary Data 1 [file mmc1.docx]

Design, implementation and evaluation of non-contact in vivo confocal microscopy for capturing the human corneal endothelium from the central to near-limbal regions

Karsten Sperlich^1,2^, Alois Gottschlich^1^, Karsten Winter^3^, Florian Worsch^1,2^, Oliver Stachs^1,2^ and Sebastian Bohn^1,2^

Corresponding author: Karsten Sperlich, Ph.D.

karsten.sperlich@uni-rostock.de

Doberaner Str. 140, 18057 Rostock, Germany

^1^Department of Ophthalmology, Rostock University Medical Center, 18057 Rostock, Germany

^2^Department Life, Light & Matter, University of Rostock, 18059 Rostock, Germany

^3^Institute of Anatomy, University of Leipzig, 04103 Leipzig, Germany

**Supplementary Material**


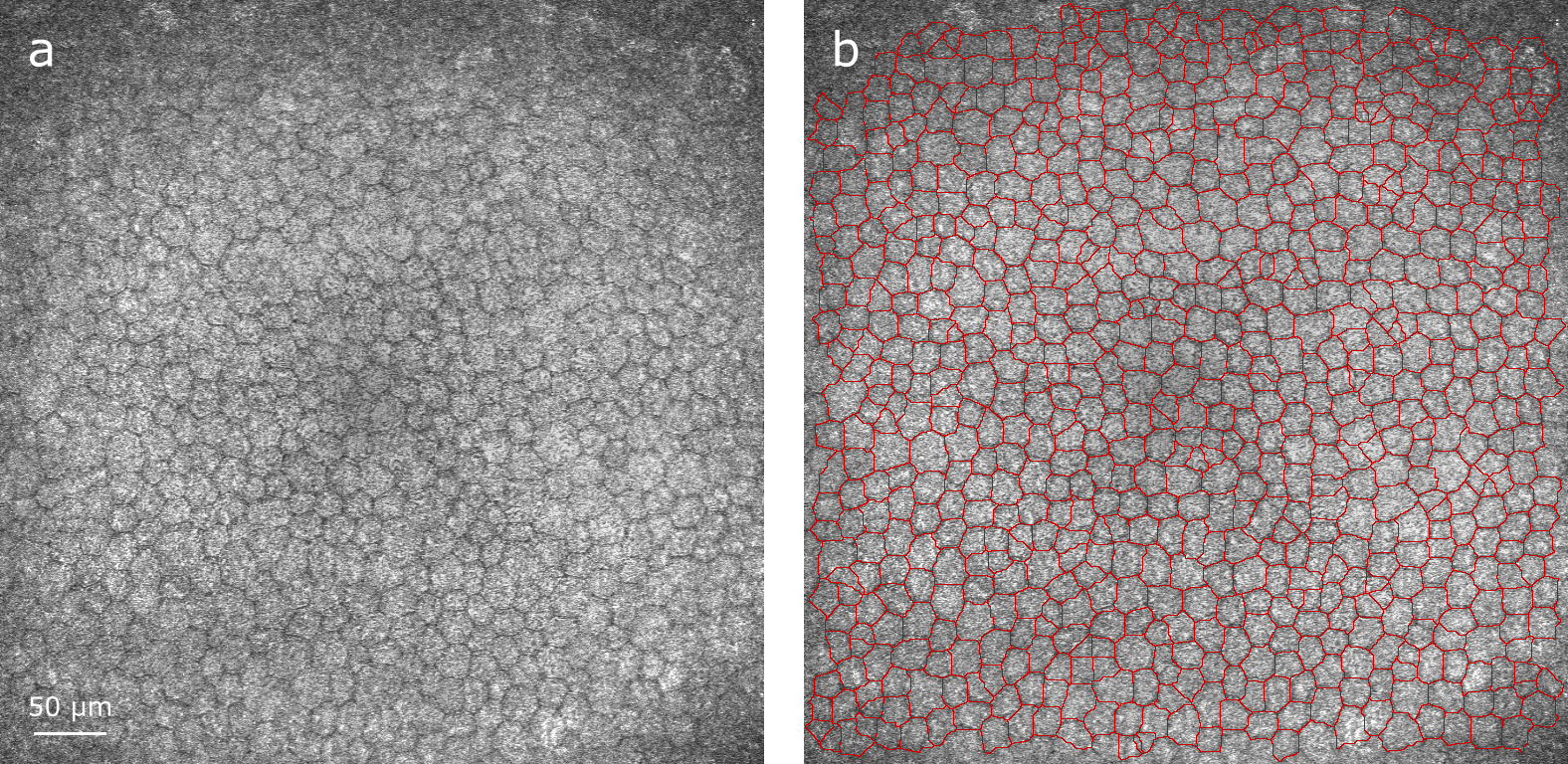


*Figure S1: Non-contact in vivo confocal microscopy of the central corneal endothelium of a 62-year-old female healthy volunteer acquired with the modified SPECTRALIS setup: (a) original image and (b) the same image with automated cell segmentation (red overlay). The segmented area was 0.262 mm*² *(88.8 % of FOV). The automated parameters were: mean cell diameter (22.4 µm), mean cell area (324 µm²), endothelial cell density (3091 cells/mm²), polygonality (5.4), mean neighbor count (5.9) and cell count (809).*


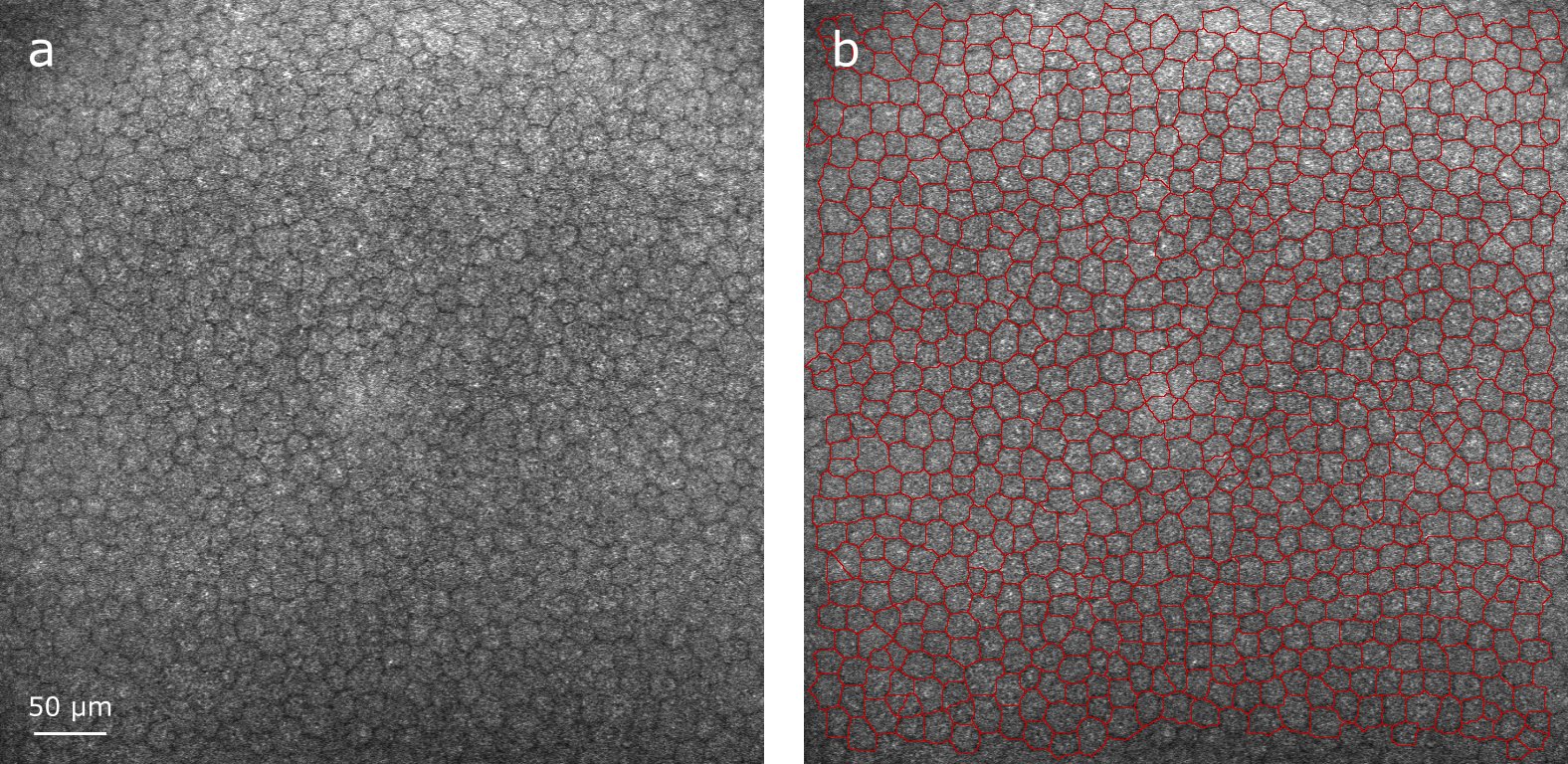


*Figure S2: Non-contact in vivo confocal microscopy of the central corneal endothelium of a 27-year-old male healthy volunteer acquired with the modified SPECTRALIS setup: (a) original image and (b) the same image with automated cell segmentation (red overlay). The segmented area was 0.263 mm*² *(89.2 % of FOV). The automated parameters were: mean cell diameter (21.7 µm), mean cell area (306 µm²), endothelial cell density (3265 cells/mm²), polygonality (5.6), mean neighbor count (5.9) and cell count (857).*


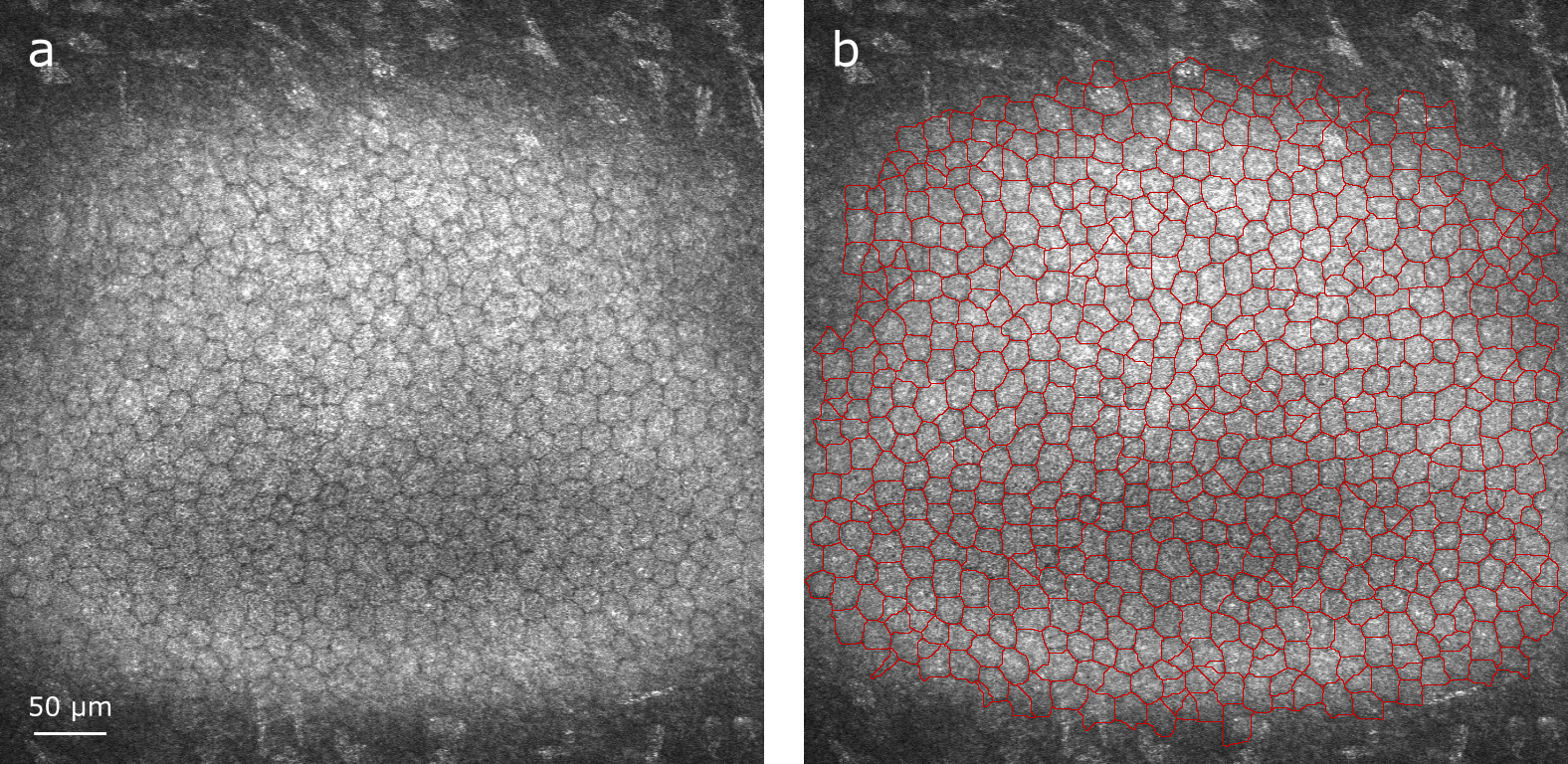


*Figure S3: Non-contact in vivo confocal microscopy of the central corneal endothelium of a 22-year-old male healthy volunteer acquired with the modified SPECTRALIS setup: (a) original image and (b) the same image with automated cell segmentation (red overlay). The segmented area was 0.213 mm*² *(72.2 % of FOV). The automated parameters were: mean cell diameter (22.4 µm), mean cell area (320 µm²), endothelial cell density (3129 cells/mm²), polygonality (5.4), mean neighbor count (5.8) and cell count (667).*


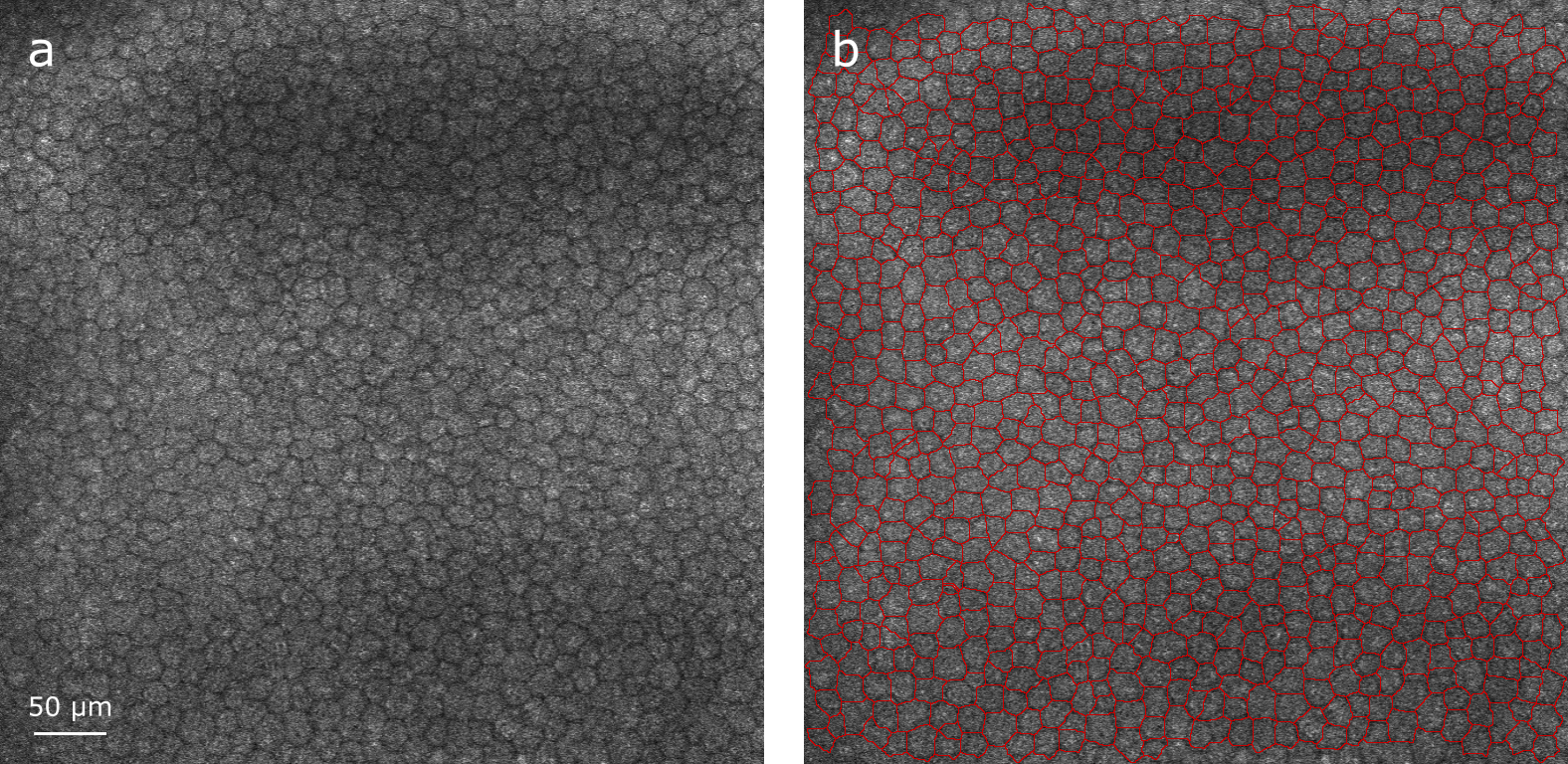


*Figure S4: Non-contact in vivo confocal microscopy of the central corneal endothelium of a 21-year-old female healthy volunteer acquired with the modified SPECTRALIS setup: (a) original image and (b) the same image with automated cell segmentation (red overlay). The segmented area was 0.268 mm*² *(90.9 % of FOV). The automated parameters were: mean cell diameter (20.6 µm), mean cell area (277 µm²), endothelial cell density (3616 cells/mm²), polygonality (5.5), mean neighbor count (5.9) and cell count (969).*


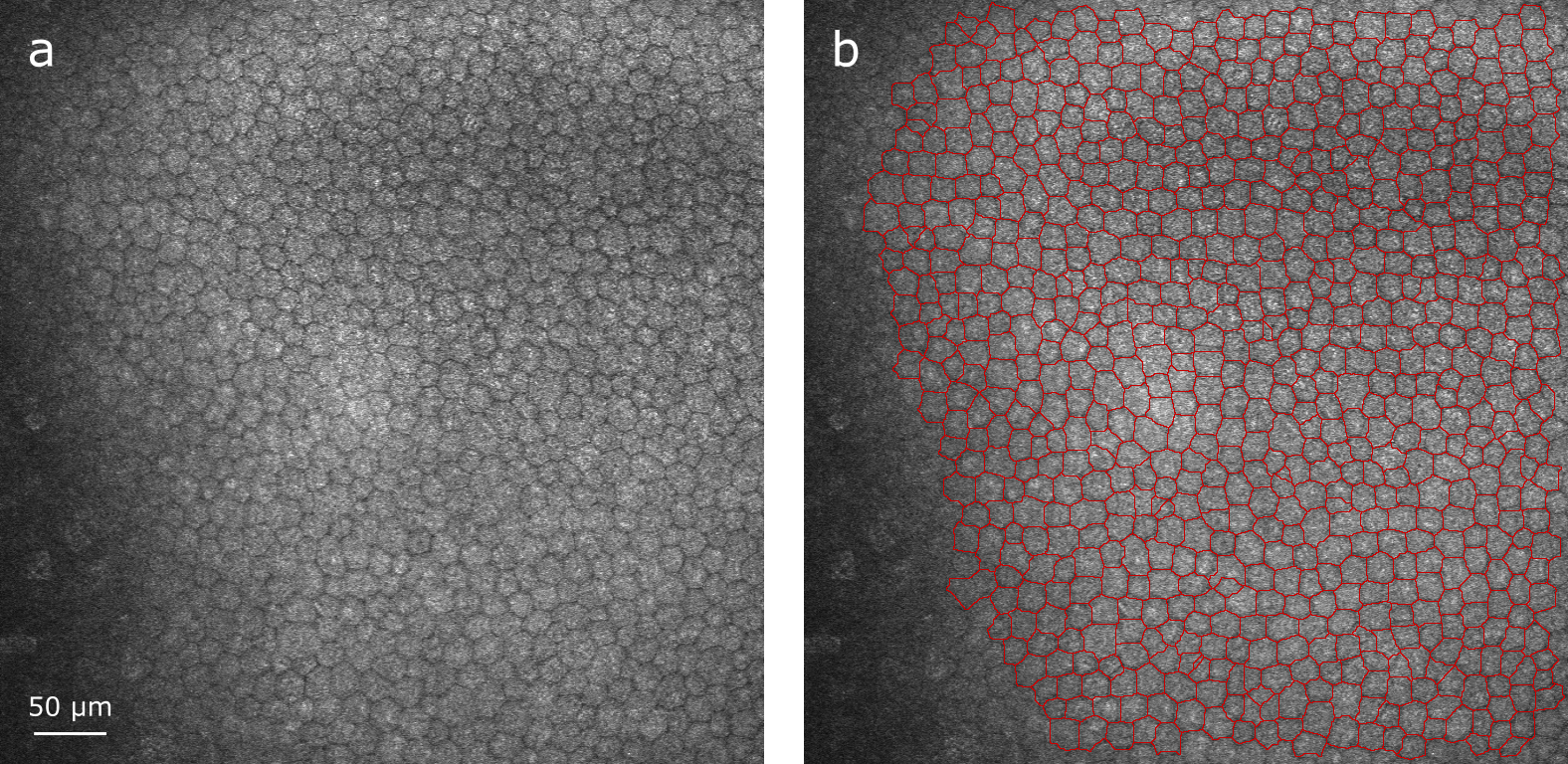


*Figure S5: Non-contact in vivo confocal microscopy of the central corneal endothelium of a 31-year-old male healthy volunteer acquired with the modified SPECTRALIS setup: (a) original image and (b) the same image with automated cell segmentation (red overlay). The segmented area was 0.225 mm*² *(76.3 % of FOV). The automated parameters were: mean cell diameter (20.7 µm), mean cell area (284 µm²), endothelial cell density (3525 cells/mm²), polygonality (5.6), mean neighbor count (5.9) and cell count (792).*
